# The cost-effectiveness of three methods of disseminating information to improve medical male circumcision in Uganda

**DOI:** 10.1371/journal.pone.0195691

**Published:** 2018-04-19

**Authors:** Edward I. Broughton, Esther Karamagi, Angella Kigonya, Anna Lawino, Lani Marquez, Sarah Smith Lunsford, Albert Twinomugisha

**Affiliations:** 1 University Research Co., LLC, Chevy Chase, Maryland, United States of America; 2 USAID Applying Science to Strengthen and Improve Systems (ASSIST) Project, Chevy Chase, Maryland, United States of America; 3 International Health Department, Johns Hopkins School of Public Health, Baltimore, Maryland, United States of America; 4 University Research Co., LLC, Kampala, Uganda; 5 EnCompass, LLC, Rockville, Maryland, United States of America; Central University of Tamil Nadu, INDIA

## Abstract

**Background:**

Uganda is working to increase voluntary medical male circumcision (VMMC) to prevent HIV infection. To support VMMC quality improvement, this study compared three methods of disseminating information to facilities on how to improve VMMC quality: M—providing a written manual; MH—providing the manual plus a handover meeting in which clinicians shared advice on implementing key changes and participated in group discussion; and MHC—manual, handover meeting, and three site visits to the facility in which a coach provided individualized guidance and mentoring on improvement. We determined the different effects these had on compliance with indicators of quality of care.

**Methods:**

This controlled pre-post intervention study randomized health facility groups to receive M, MH, or MHC. Observations of VMMCs performance determined compliance with quality indicators. Intervention costs per patient receiving VMMC were used in a decision-tree cost-effectiveness model to calculate the incremental cost per additional patient treated to compliance with indicators of informed consent, history taking, anesthesia administration, and post-operative instructions.

**Results:**

The most intensive method (MHC) cost $28.83 per patient and produced the biggest gains in history taking (35% improvement), anesthesia administration (20% improvement), and post-operative instructions (37% improvement). The least intensive method (M; $1.13 per patient) was most efficient because it produced small gains for a very low cost. The handover meeting (MH) was the most expensive among the three interventions but did not have a corresponding positive effect on quality.

**Conclusion:**

Health workers in facilities that received the VMMC improvement manual and participated in the handover meeting and coaching visits showed more improvement in VMMC quality indicators than those in the other two intervention groups. Providing the manual alone cost the least but was also the least effective in achieving improvements. The MHC intervention is recommended for broader implementation to improve VMMC quality in Uganda.

## Introduction

Many countries in sub-Saharan Africa, including Uganda, are working to significantly increase voluntary medical male circumcision (VMMC) prevalence as a prevention strategy against HIV infection [1]. Part of facilitating this initiative is to provide free circumcision services that are safe and of high quality [2–4], which are important factors in creating demand for the procedure [5, 6]. The US Agency for International Development (USAID) has supported the application of improvement methods in 100 clinics offering VMMC throughout Uganda with the goal of improving the performance of service delivery. To support improvement of VMMC services in all sites offering the service nationwide, it is of interest to the Ministry of Health and donors to spread improvements to the system of VMMC care as efficiently as possible.

Interventions that aim to improve health care service delivery are generally first implemented in a small number of sites in a circumscribed geographical region [7]. They are tested over time to determine their effectiveness in improving quality and efficiency. Successful interventions are then spread over a wider geographical range to include many more and often all health care sites in a jurisdiction. Scaling up the intervention involves dissemination of methods and change ideas to improve health care delivery and empowering front-line health workers to adopt them. Successful spread is critical to health system improvement and often relies on fewer inputs from outside groups and fewer overall resources per facility covered than the initial intervention [8]. This is especially the case in low- and middle-income settings.

Methods for disseminating health system improvement principles and activities can be accomplished by various modes of knowledge management (KM)–the development, sharing, and effective use of organizational knowledge developed in the piloting phase of the intervention. However, there is very little research on the effectiveness and efficiency of different KM methods that are used to bring improvement principles and practices to facilities as part of a scale-up phase of interventions to improve quality of care in health facilities.

This study examined the effectiveness and cost-effectiveness of three modes of disseminating information to facility-based health care workers in Uganda to enable them to implement changes to improve the functioning of VMMC services.

## Intervention

The three methods of dissemination, which encompassed activities implemented in an additive way, were:

### M (Manual)

Distribution of a well-designed written manual developed based on synthesis of what the initial sites learned about improving different aspects of VMMC. One copy of the manual was delivered to each facility by a staff member of the improvement technical assistance project. That person met with facility staff in a meeting lasting under one hour to provide an overview of the content of the manual (its structure and organization), share the table of contents of the manual, and briefly describe the main sections so that facility staff were aware of the information available in the manual.

### MH (Manual and handover meeting)

Provision of the manual plus participation of three staff from the facility in a two-day handover meeting in Kampala, the capital, with clinicians from facilities that had successfully undergone changes to improve VMMC services from the demonstration intervention. Clinicians shared their advice on how to implement key changes and participated in small group conversations with the representatives of the study facilities. Staff from implementing partners supporting the sites and Ministry of Health (MOH) coaches who had experience supporting sites to improve VMMC services also participated. Staff of the improvement technical assistance project facilitated the meeting. A few days before the handover meeting, each participating site was visited to ask facility staff about their learning needs and any specific questions they had to be addressed in the handover meeting.

### MHC (Manual, handover meeting and coaching visits)

Provision of written materials, the handover meeting, and coaching support were provided. Coaching support consisted of three site visits by a trained circumciser experienced in providing guidance and mentoring on improvement methods beginning 6 to 8 weeks after the handover meeting to follow up on how the site was implementing the key changes. These coaches also participated actively in the handover meeting and provided contact information to encourage sites to contact her or him by phone or email if they encountered problems. The coach also made phone calls to the facility before and after each coaching visit to provide continued virtual support. Three coaching visits were made over a four-month period.

## Research objectives

We sought to determine the different effects that these three methods of disseminating information about improving VMMC care in terms of compliance with indicators of quality of care. We compared changes in these quality indicators from the pre-intervention period to the post-intervention period in health care sites that had been exposed to the three information dissemination methods.

We also sought to estimate the incremental cost-effectiveness ratio of the different information dissemination methods compared to one another in terms of expenditure per unit of patient care improvement achieved in terms of the quality of care indicators. While results would have been easier to understand if we could have used outcome indicators such as post-surgery adverse events averted, it was not possible because of practical limitations on the follow-up of patients over a longer period. Therefore, we used process measures as proxies. This was considered appropriate given that the main goal of the study was to compare among the three information dissemination methods used and not compare the effects seen to interventions outside this activity.

## Methods

### Study design and sampling

This is a controlled pre-post intervention study randomized at the level of groups of health facilities. Selection of facilities to participate in the study was done by the following steps:

1. The funder provided a list of sites where the intervention was to be implemented. This was based on which facilities were receiving support from the funder.
2. We removed from the list all health centers (HC) level III (the lowest level unit providing VMMC services in Uganda) sites and all other sites that averaged fewer than 50 VMMCs per month. Study sites included: (1) general hospitals (which provide preventive, promotive, maternity, and curative in-patient health services); (2) regional referral hospitals that provide specialist services, teaching and research in addition to those for general hospitals; and (3) health centers level IV that provide preventive, promotive, rehabilitative, and curative services, including emergency obstetric care with cesarean section and blood transfusion [9].
3. Facilities that had substantial experience with external support for improvement interventions previously through other projects were eliminated.
4. We ensured that there was representation from five languages and from five regions of the country.
5. The remaining list of facilities was divided into three arms so that there was representation of HC IVs and hospitals and all regions in all arms. This gave three arms matched on characteristics considered important to the outcome variables in this study.
6. Each of the three arms was randomly assigned to one of the three information dissemination methods being investigated.

Patient participants were 18 years of age or older and selected randomly all from those who received VMMC services at one of the selected health care facilities. They were stratified into time blocks and then selected randomly by assigning identification numbers using the Excel random number function. If the number of eligible patients was not greater than the number sought for the sample, we took all eligible patients receiving VMMC services that the facility.

Patients whose procedures were observed were randomly selected from all those receiving care from the participating facility on the days of observation and who provided informed consent.

The sample size was based on detecting a difference of 15% on the dichotomous variables (compliance or non-compliance with a given quality indicator) from the baseline period prior to the intervention with a power of 0.8, alpha of 0.05, and a design effect of 2.0 to account for facility clustering. This gave a sample size of about 370 for each group of observations, for a total of 2,220 observations.

### Research ethics

The study protocol, informed consent forms, and data collection instruments were approved by the Institutional Review Boards of University Research Co., LLC in the US (January 31, 2014) and Mildmay Research and Ethics Committee (March 20, 2014). Approval was also granted by the Uganda National Council for Science and Technology (Research Number 3528, approved July 1, 2014) and from the individual participant health facilities.

### Data collection

Trained research assistants were deployed as observers to the participating facilities throughout Uganda. After obtaining informed consent from clinicians and verbal consent from patients, they observed the conduct of the entire procedure from an inconspicuous vantage point. They recorded compliance with each of the criteria listed in Table 1 in a notepad to be transferred to a computer spreadsheet the same day. The observers were well versed in the recommended manner of performing the procedure and were asked only to interfere if they witnessed something that could endanger the safety of the patient or staff. No such intervention on the part of the observers was required during the data collection.

**Table 1:** Quality of care criteria used to determine improvement in VVMMC services.

*Domain*	Criteria
Consent	1. Provider checks client understanding of risks/complications before signing consent form
History taking	2. Current general health
	3. STI history (current symptoms—dysuria, ulcers, discharge, pain)
	4. What medications client is taking
	5. What medication allergies are known
	6. History of hemophilia, bleeding disorders or anemia
	7. Previous surgeries and reaction to anesthesia
	8. Whether the client has erectile dysfunction / other sexual function problem
	9. Completed client history section of SMC Client Record
Anesthesia administration	10. Drapes client to expose genitalia surgical site only
	11. Administers correct anesthesia dose (max 3mg/kg) at base of penis (eleven & one o’clock then ring block)
	12. Waits 3–5 min for anesthetic to take effect
Post-operative instructions	13. Shows how to remove and reapply strapping before and after urinating
	14. Avoid intercourse and masturbation for 6 weeks
	15. Wear clean, loose fitting underwear which should be changed each day
	16. Do not wet the dressing for the first 2 days
	17. After 2 days wash the genitalia with non-medicated soap and lukewarm water
	18. Remember to come for follow-up visit after 2 days and one week
	19. Recognize & return if signs of complications (excessive bleeding, dysuria, excessive pain, swelling, oozing pus)
	20. Rest for 2 days
	21. Reinforces HIV prevention messages
	22. Ensures client knows where to go if complication arise and has contact phone number
	23. Provides post-op SMC leaflet
	24. Gives next appointment date (48 hour and 7 day follow-up)
	25. Gives information on how to manage post-operative penile erections
	26. Provides client with condoms

Costs were considered from the perspective of the program implementer (the improvement technical assistance project), and data were collected from the accounting records of the implementing organization. These costs included all expenditures of the implementer for the three information dissemination methods. Items included were: printing and distribution costs for the manual; transport and accommodation costs for participants and the presenters, venue rental and meeting materials, and salaries for presenters for the handover meeting; and transportation, accommodation, and salaries for the coaches for the coaching sessions. Costs to the MOH for staff time and any other additional resources consumed in the activities were not included. This is because health workers’ performance of VMMC services were considered part of their normal work activities, and they were not paid any additional award for their contributions to the data collection.

### Data analyses

Data were analyzed by first grouping the 26 quality of care criteria into the four domains of informed consent, history taking, anesthesia administration, and post-operative instructions. We then determined for each observation conducted whether there was:

1. Compliance with the one indicator for obtaining informed consent,
2. Compliance with six or more of the eight history-taking indicators,
3. Compliance with two or more of the three anesthesia indicators, and
4. Compliance with 12 or more of the 14 post-op instructions indicators.

These thresholds were determined as reasonable approximations of good quality VMMC by the clinical experts on the study team. We then determined the proportion of patient observations that achieved that level of performance before and after the intervention for each study group. We determined the increase seen from before the intervention to after the end of the intervention. We used these inputs for the cost-effectiveness model. The models calculated the incremental cost per additional patient procedure observed to reach those levels of compliance.

A full list of the variables is shown in Table 1 but for the cost-effectiveness analyses, they were considered in their domains (Column 1). For example, we determined what proportion of clients were given adequate discharge instructions in the three groups before and after the intervention. In the analysis, we controlled for patients’ ages and other demographic characteristics that were potential confounders.

A decision tree was used to determine the incremental cost-effectiveness ratios of the three information dissemination methods each compared with the base case. The base case was represented by the pre-intervention level of compliance with quality indicators. We used the program funder’s perspective (USAID) for determination of costs and effects. The basic formula used was:

$$\begin{array}{l} \text{Incremental}\;\text{cost}\;\text{effectiveness}\;\text{ration}\;\left(\text{ICER}\right)=(\text{Difference}\;\text{in}\;\text{costs}\;\text{between}\;\text{intervention}\\ \left[\text{M},\;\text{MH}\;\text{or}\;\text{MHC}\right]\;\text{and}\;\text{the}\;\text{baseline}\;\text{of}\;\text{no}\;\text{intervention})÷(\text{difference}\;\text{in}\;\text{effects}\;\text{of}\;\text{improvement}\\ \text{indicator}\;\text{between}\;\text{intervention}\;\left[\text{M},\;\text{MH}\;\text{or}\;\text{MHC}\right]\;\text{and}\;\text{the}\;\text{baseline}\;\text{of}\;\text{no}\;\text{effects}) \end{array}$$

## Results

There was improvement in the indicators of quality of VMMC services in three of the four domains for all three intervention groups. Slight decreases were noted in compliance with anesthesia standards in the M and MH groups. Improvements were much higher in the MHC group among all but the signed consent indicator: 35% for history-taking, 20% for anesthesia, and 37% for post-op instructions (Table 2).

**Table 2:** Differences in indicators between pre- and post-intervention periods, costs and cost-effectiveness of the three interventions compared to no intervention.

		Manual Only		Handover meeting		3 Coaching visits	
		Cost	Patients	Cost	Patients	Cost	Patients
		$5000	2130	$45200	1494	$6500	808
Obtaining informed consent	% improvement	5		6		0	
	ICER	22.56		346		577	
	95% CI	8–37		108–583		377–787	
History taking	% improvement	18		0		35	
	ICER	7.52		NA		75.87	
	95% CI	0–37.6				57.67–115.33	
Anesthesia	% improvement	-5		-5		20	
	ICER	NA		NA		320.37	
	95% CI					180.21–1441.67	
Post-op instructions	% improvement	10		24		37	
	ICER	22.58		230.77		240.28	
		0–214.75		109.02–676.98		137.3–961.11	

For the differences among the intervention groups using linear regression, the MHC group showed significantly better improvement than the M and MH groups in the history, anesthesia, and post-operative instructions indicators (Table 3). Variables in the linear regression equation used were time period (pre- or post-intervention), an indicator for the intervention group or control, the interaction term for time period and intervention/group, and facility, to account for clustering at the facility level. The reference was the second terms for each column in Table 3 (M, M, and MH, respectively).

**Table 3:** Comparison of changed in quality indicators from the three interventions.

Indicator	MH-M diff			MHC-M diff			MHC-MH diff		
	%	95% CI		%	95% CI		%	95% CI	
Signed Consent	2	-3	6	-4	-10	1	-6	-10	1
History	16	-23	-10	17	9	25	34	27	40
Anesthesia	0	-3	3	25	21	29	25	2	29
Post-op instructions	14	10	18	26	20	32	12	8	17

The costs of the interventions were $5,000 for M, an additional $45,200 for MH, and a further $6,500 for the MHC intervention groups. The interventions were conducted in facilities where 2,130, 1,494, and 808 VMMCs were performed. The cost of receiving the M intervention was $1.13 per patient receiving services at those facilities. The cost of the MH intervention was $20.77 per patient, and the cost of the MHC intervention was $28.83 per patient. For compliance with informed consent compared to business-as-usual, the cost-effectiveness of providing the M intervention to improve quality of service delivery is $22.56 per additional person provided services to compliance (95% CI: 8.06 to 37.06). The cost-effectiveness of providing the MH intervention was $346.21 per additional person provided informed consent (95% CI: $108.80 to 583.20). The cost-effectiveness of providing the MHC intervention to facilities is $576.67 per additional person provided informed consent (95% CI: $377.26 to 786.87) (Table 2).

The NA in the table means that there was no improvement in the indicator from before to after the intervention; therefore, the cost-effectiveness analysis was not calculated.

The cost-effectiveness results are additive. This means the improved compliance in obtaining informed consent, improved history taking, and improved compliance to standards of anesthesia and to post-operative instructions were achieved in the one program with its total cost. Therefore, the result can be stated as:

- For every $10,000 of expenditure on providing manuals to promote improvement in circumcision services, there were 443 additional patients who gave appropriate informed consent, 1330 additional patients who had at least 75% or their history taken, and 443 additional patients who were given at least 75% of the mandated post-op instructions.
- For every $10,000 of expenditure on providing manuals and a handover meeting for facility staff to promote improvement in circumcision services, there were 29 additional patients who gave appropriate informed consent, and 43 additional patients who were given at least 75% of the mandated post-op instructions.
- For every $10,000 of expenditure on providing manuals, a handover meeting, and three coaching visits to promote improvement in circumcision services, there were 17 additional patients who gave appropriate informed consent, 132 additional patients who had at least 75% or their history taken, 31 patients who received anesthesia according to evidence-based guidelines for at least two of the three indicators, and 42 additional patients who were given at least 75% of the mandated post-op instructions.

## Discussion

From these results, the MHC intervention was the one associated with the greatest increases in three of the four selected quality of VMMC domains from before the intervention to after it. The cost of providing the single element of the M intervention was substantively less than the other two intervention groups as expected, but the patients receiving their circumcision procedures from M group facilities actually experienced some declines in the grouped quality indicators according to the method we used to analyze them. If the overall goal of improvement is to improve compliance with all groups of indicators that exist for VMMC services, then clearly the more intensive MHC intervention is warranted. However, if funds to invest in improvement of VMMC services are limited, the M intervention has some merit because it improves informed consent and compliance with history-taking and post-operative instruction provision more efficiently than the two more intensive interventions, assuming any negative effects of suboptimal anesthesia administration could be eliminated. One may conclude that all 26 quality indicators for VMMC were selected with care and included because of their importance for patient safety, comfort, and satisfaction. Therefore, the intervention that achieves the best improvements across all indicators, namely MHC, is the one most highly recommended for implementation in this setting, even though it was a less efficient means of achieving improvement in some quality indicators. It also leaves the smallest proportion of those receiving VMMC who would get sub-standard treatment in the participating facilities.

The quality of VMMC services is very important for increasing the number of men circumcised for HIV prevention, and improvement interventions have been implemented successfully in other settings [10–12]. Of particular importance in recent times is the focus on delivering such services in sub-Saharan Africa at large scale while still maintaining high quality of services to reduce adverse events and encourage greater uptake among the target population [13–16]. While the study was not powered to detect differences in the effects among different regions, the uniformity of results and previous experience suggest the findings are generalizable across Uganda [10]. Results from this study may be especially relevant in many settings in Africa to guide decision-making for maximal public health benefit.

We could find no other recent studies in low- or middle-income settings that examined the relative cost-effectiveness of different methods of disseminating information to improve health worker performance. One study in Malawi compared a control and two training programs for lay health workers to improve their competence with managing tuberculosis patients. It did not have a cost component but aimed to address a similar question [17]. It found no significant difference in patient outcomes associated with the different knowledge translation methods.

Weaknesses in this study include the lack of a single valid measure for the quality of VMMC services or measures of a clinical outcomes such as the occurrence of adverse events that we could have used rather than the multiple proxy indicators that we used from necessity. While using such a multiplicity of measures makes interpretation of the results more complex, we believe it still gives a valid indication of the effectiveness of each of the intervention groups used here.

The way we analyzed the individual indicators to give the four domains of quality was informed by those with experience in the field of VMMC improvement interventions rather than a widely accepted method, which is unavailable. Given that the goal of the study was to compare the three interventions to each other rather than compare this intervention to other health interventions, we found this method acceptable.

Another weakness was the limited number of combinations of intervention elements we tested. It would have provided more useful information had we combined the manual distribution with only coaching visits and the handover meeting only and the coaching visits only as other intervention types to test for effectiveness. The manual and coaching visits intervention would have been of particular interest because it would have been inexpensive relative to interventions including the handover meeting. But resources and the number of facilities available for recruitment into the study made this infeasible.

## Conclusion

This study found that health workers in facilities that received a printed manual on improvement methods and participated in both a handover meeting and coaching visits showed more improvement in process indicators of quality of VMMC. The handover meeting was the most expensive element among the three interventions but did not appear to have a correspondingly large additive effect on quality indicator improvement. Providing the manual alone was the least costly method of disseminating information about how to improve health services, but it was also the least effective in achieving improvements.
